# Determination of volume averaging correction factors using an elliptical absorbed dose model for Gamma Knife Perfexion

**DOI:** 10.1002/acm2.14109

**Published:** 2023-08-25

**Authors:** Nikola Šegedin, Hrvoje Hršak, Sanja Dolanski Babić, Slaven Jurković

**Affiliations:** ^1^ Department for Physics and Biophysics School of Medicine University of Zagreb Zagreb Croatia; ^2^ Department for Medical Physics University Hospital Centre Zagreb Zagreb Croatia; ^3^ Department for Medical Physics and Biophysics Faculty of Medicine University of Rijeka Rijeka Croatia; ^4^ Department for Medical Physics and Radiation Protection University Hospital Centre Rijeka Rijeka Croatia

**Keywords:** elliptical dose model, Gamma Knife Perfexion, small field dosimetry, volume averaging correction factors

## Abstract

**Purpose:**

The purpose of this study is to calculate volume averaging correction factors for detectors used in the dosimetry of Gamma Knife's narrow photon beams, and to determine the impact of volume averaging on the field output correction factor.

**Methods:**

Simulations of different Gamma Knife fields were done using elliptical dose model formalism with newly introduced fit functions. To determine volume averaging correction factors a calculation of the absorbed dose over the volume of the detector was performed. The elliptical dose model was tested with respect to absorbed dose distribution for different volumes and compared with the calculations of Leksell GammaPlan v.11.3.1.

**Results:**

The largest differences in absorbed dose calculated by the elliptical model and Leksell GammaPlan are 2.25%, 1.5%, and 0.6% for 16, 8, and 4 mm field sizes, respectively. Volume averaging correction factors were determined for six ionization chambers, five semiconductor detectors, a diamond, and two plastic scintillator detectors. In general, for all examined detectors the impact of volume averaging is more pronounced for smaller field sizes. All studied ionization chambers had a larger volume than other detectors, therefore the volume averaging correction factors for ionization chambers are larger for all investigated field sizes. Besides the fact that plastic scintillator detectors can be considered tissue‐equivalent, volume averaging correction factor should be applied.

**Conclusion:**

Volume averaging correction factors for different detectors are determined and suitable detectors for dosimetry of Gamma Knife's narrow photon beams are recommended. It is shown that volume averaging has a dominant contribution to a field output correction factor.

## 1 INTRODUCTION

Stereotactic radiosurgery (SRS) using Leksell Gamma Knife Icon (GK, Elekta, Stockholm, Sweden) built on the Perfexion platform is a well‐established modality that utilizes 192 narrow‐collimated Co‐60 beams to treat a multitude of well‐defined intracranial lesions.^1^, ^2^ Application of such narrow photon beams enables one to deliver a high absorbed dose to a tumor volume to maximize tumor control while minimizing normal tissue complication probability. Gamma Knife Perfexion (GK) has three different circular collimators with diameter projection at the isocenter of 16, 8, and 4 mm, creating 3D elliptical fields, all of which can be considered small fields. Such absorbed dose distributions are characterized by a high dose gradient (up to 80%/mm) and loss of lateral charged particle equilibrium (LCPE) which complicates the selection of detector for accurate referent and relative dosimetry.

**FIGURE 1:**
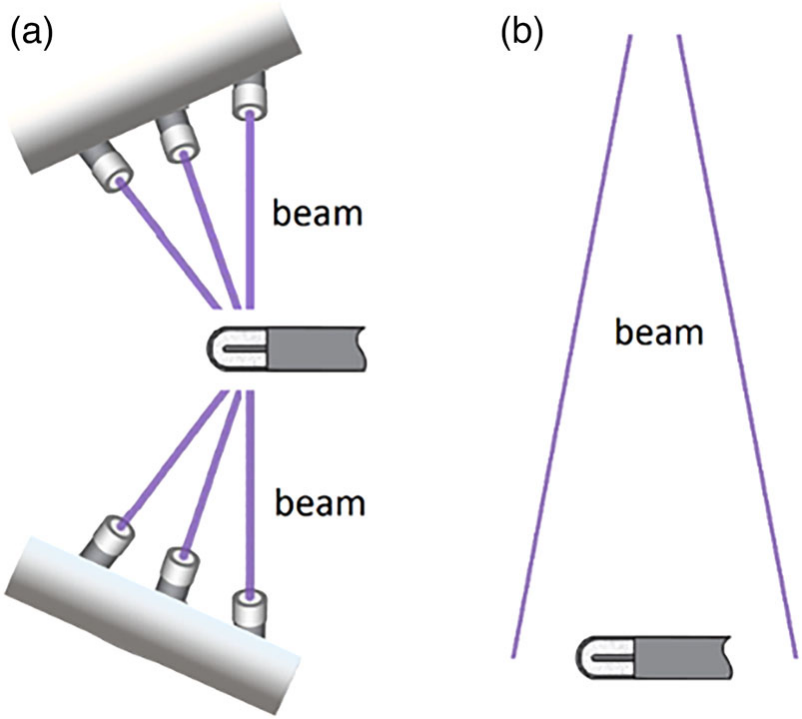
An illustration of beams incident on the detector for (a) Gamma Knife Perfexion, and (b) Linear accelerators, CyberKnifes, and Tomotherapy units.

For small field dosimetry, it is crucial to determine the field output correction factor $k_{Q_{msr},\;Q_{clin}\;}^{f_{msr},\;f_{clin}}$ to be able to correct the detector's reading and account for the abovementioned nonequilibrium conditions in machine‐specific reference f_msr_ (msr) and clinical fields f_clin_ (clin) with beam qualities Q_msr_ and Q_clin_. Together with the field output factor (OF) $\Omega_{Q_{msr},\;Q_{clin}}^{f_{msr},f_{clin}}$ determination, these were the focus of investigation performed by different groups.^2^, ^3^, ^4^, ^5^, ^6^, ^7^, ^8^, ^9^, ^10^, ^11^ OF and field output correction factor determination for different detectors can be performed by measurements or with Monte Carlo (MC) simulations. In general, field output correction factors are a product of four different perturbations in the small field; ratios of water‐to‐detector‐medium stopping powers in the clinical and machine‐specific reference field, volume averaging, fluence, and of spectral perturbations in clinical and machine‐specific reference fields.^12^ Volume averaging of a measured detector's response is a known problem in small photon beam dosimetry (SPBD) which leads to underestimation of an absorbed dose on the field's central axis and widening of the dose profile. Volume averaging correction factor is defined as the dose averaged over a volume of water where water volume coincides with the volume of a detector, and for the determination of field output factors, it will be one of the limiting factors for the choice of the detector.^13^ International Atomic Energy Agency (IAEA) in Technical report series no. 483 Code of Practice (TRS‐483 CoP) states that the selection of detectors based on the volume averaging correction should be such that it is limited by $0.95\leq {\left(k_{vol}\right)}_{Q_{msr,\;clin}}^{f_{msr,\;clin}\;}\leq 1.05$. Since the radiation source on GK is Co‐60, the beam quality in msr and clinical fields is the same, they will be denoted as Q_0_ from now on. Although volume averaging of a measured detector's response is a subject of much dosimetry research, they are concentrated primarily on narrow x‐ray beams (linear accelerators, CyberKnifes, and Tomotherapy units) where the beam is incident on the detector from a single direction.^14^, ^15^, ^16^, ^17^ For these devices volume averaging correction factor can be calculated using already available formalism.^13^, ^18^ However, no volume averaging correction factors for commonly used detectors for Gamma Knife dosimetry have been available in the literature so far. Rarely published research on this topic involved custom‐printed isodose‐shaped scintillator detectors.^19^ The lack of research can be primarily contributed to the complex geometry of the Gamma Knife radiation delivery system where multiple beams are incident on the detector from numerous different directions creating an elliptical absorbed dose distribution and making the calculations of volume averaging correction factor more complex. Accurate determination of volume averaging correction factors in such narrow beams involves modification of current formalism from 2D to 3D approach and integration of the absorbed dose distribution over detector volume. An illustration of the difference in beam geometry of Gamma Knife Perfexion and other treatment units is shown in Figure 1.

The aim of this work was the determination of volume averaging correction factors for fourteen detectors that are commonly used for dosimetry of Gamma Knife Perfexion photon beams. For this it is necessary to determine the absorbed dose over the detector's effective volume as shown in Equation (1). Using an elliptical dose model, by fitting normalized dose profiles along the ellipse major axis to a newly introduced function (Equations 2 and 3), an analytical expression that determines the relative dose as a function of distance from the isocenter in 3D space $D(\vec{r})$ (Equation 4) was developed. The elliptical dose model was previously used for an independent dose verification for GK model C treatment planning with satisfactory results.^20^ Nonetheless, we assessed the model's accuracy with respect to the absorbed dose distribution for different volumes. Finally, with an analytical expression, a numerical integration (Simpson's method) was performed over the detector's volume to determine volume averaging correction factors.

## 2 METHODS

Volume averaging correction factor is defined as a ratio of actual absorbed dose D and mean absorbed dose D_mean_ averaged over the detector's volume V:

(1) $$k_{vol\;}=\frac{D}{D_{mean}}\;=\frac{D}{\frac{\int_{V}D\left(\vec{r}\right)d^{3}\vec{r}}{V}}\;=\frac{V}{\int_{V}D{\left(\vec{r}\right)}_{norm}\;d^{3}\vec{r}}\;$$

where $D{\left(\vec{r}\right)}_{\mathrm{norm}}$ is normalized dose distribution. Due to the volume averaging effect coupled with the fluence and spectral perturbations the detector's response will be different in machine‐specific reference and clinical fields. Therefore, the detector reading has to be corrected with the product of these perturbations resulting in the field output correction factor. To calculate volume averaging correction factors of a given detector (Equation 1) it is necessary to be aware of the detector's exact effective volume and the total absorbed dose over this volume. For this, an analytical description of an elliptical dose model for Gamma Knife Perfexion was implemented with normalized dose profiles, calculated using Monte Carlo and provided by the vendor, fitted to an analytical expression along principal GK axis (x, y and z) with function:

(2) $$D_{k}\;\left(k\right)=\sum_{i=1}^{n}A_{i}\mathrm{erf}\left(\frac{k+a_{i}}{b_{i}}\right)+C_{0}\;\;$$

where *k* represents the distance from the center of dose profiles on each principal axis (x, y and z), *Ai*, *ai*, *bi and C_0_* are fitting parameters and the *erf(z)* is an error function. The error function is a function of a complex variable (or Gauss error function) and it is defined as:

(3) $$\mathrm{erf}\left(z\right)=\frac{2}{\sqrt{\pi }}\;\int_{0}^{z}e^{-t^{2}}dt$$

For x and y‐axis dose profiles for all field sizes *n* = 4, for the z‐axis of 16 and 8 mm dose profiles *n* = 5, and *n* = 2 for the 4 mm z‐axis dose profile. In building a model, it is assumed that dose profiles for all field sizes on the x and y principal axis are perfectly symmetrical with respect to the z‐axis, that is, *f(x,y) = f(‐x,‐y)*, consequently only positive off‐axis values were considered when fitting. On the other hand, z‐axis dose profiles are asymmetrical with respect to the XY plane and both positive and negative off‐axis values must be considered when fitting. The origin of this asymmetry is the geometry of the GK collimator system where Co‐60 sources are symmetrically distributed around the z‐axis but not around the x and y‐axis. Asymmetry is most pronounced for the 16 mm field and it is gradually reduced by decreasing the field down to 4 mm as shown in Figure 3.

In an elliptical dose model normalized absorbed dose value $D(\vec{r})$ at some point in space $r_{2}=\;\left(x_{2},\;y_{2},\;z_{2}\right)$ with respect to the center of dose distribution at point $r_{1}=\left(x_{1},\;y_{1},\;z_{1}\right)\;$ can be determined as^20^:

(4) $$D\;\left(\vec{r}\right)=\sqrt{{\left(D_{x}\frac{x_{2}-x_{1}}{\left|\vec{r_{2}}-\vec{r_{1}}\right|}\right)}^{2}+{\left(D_{y}\frac{y_{2}-y_{1}}{\left|\vec{r_{2}}-\vec{r_{1}}\right|}\right)}^{2}+{\left(D_{z}\frac{z_{2}-z_{1}}{\left|\vec{r_{2}}-\vec{r_{1}}\right|}\right)}^{2}}$$

Absorbed dose from the center to a given isodose value D_V_, enveloping an ellipsoid of volume V is calculated as dose integral over that volume, or it can be approximated with the sum by adding values of an individual dose‐at‐points inside the volume of interest:

(5) $$D_{V}=\;\int_{V}D\left(\vec{r}\right)d^{3}\vec{r}\approx \sum_{i\;=\;1}^{i\in V}D_{i}$$

For accurate volume averaging correction factor calculations, the model must accurately predict the absorbed dose for a given volume. An investigation of the difference of an absorbed dose predicted by the model with the one obtained with the treatment planning system (TPS), Leksell GammaPlan v.11.3.1 (LGP, Elekta, Stockholm, Sweden), was done for ellipsoids with different volumes starting at the 100% of absorbed dose and ending at different isodose values, eight different volume in total for all field sizes, from 100% to 20%, for example, 100%–90%, 100%–80%, …., 100%–20%. To do this, a single field was simulated for all sizes in LGP with the shot's center set at the center of Leksell's coordinate system, implying the standardized Solid Water phantom with an 80 mm radius and γ‐angle set to 90°. A total of 1700, 783, and 378 points were extracted from LGP for 16, 8, and 4 mm shots, respectively. The selection of points was randomly taken for dose values ranging from 99% to 19% of the relative dose in all spatial directions with resolution in the z‐direction of 1 mm. In MATLAB (TheMathWorks Inc., USA) an elliptical 3D dose distribution was simulated using fitted normalized dose profiles (Equation 2) for an elliptical dose model (Equation 4). To simplify calculations, dose distributions were shifted in a way that r_1_ = (0,0,0). An examination of the model's error R_V_ with respect to an absorbed dose distribution is done in a way that a set of points extracted from the LGP were entered in the model, and absorbed doses calculated by the LGP D_LGP_ and the model D_model_ are compared against each other for a different ellipsoid volume that spanned from the isocenter to a given isodose:

(6) $$R_{v}=\frac{\left|D_{LGP}-D_{model}\right|}{D_{LGP}}\;\cdot 100\%$$

**FIGURE 2:**
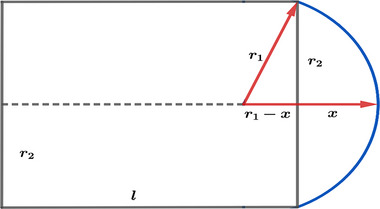
An illustration of an ionization chamber's geometry.

Volume averaging correction factors was calculated for fourteen detectors; six ionization chambers, five semiconductor detectors, a diamond detector, and two plastic scintillator detectors, all commonly used for dosimetry of GK photon beams (Table 1). This was done by simulating detector geometry inside the field by placing the detector's reference point at the field's center with the detector's long axis parallel to the GK z‐axis. Volume averaging was determined by numerically integrating absorbed dose distribution over the detector's volume using Simpson's method with one hundred steps in each spatial direction, that is, when integrating, the detector's active volume is divided into one million parts. Ionization chambers were simulated as having a cylindrical body, an electrode inside it, and a hemispherical cap on the top (Figure 2). The remaining detectors have simpler geometries of their active volume being disk‐shaped, except for the EDGE detector (Sun Nuclear, Melbourne) whose active volume is rectangular.

**TABLE 1:** A list of detectors, manufacturer, type, and volume information for which volume averaging correction factor was determined.

Detector name	Manufacturer	Type	Volume/mm^3^
Semiflex T31010	PTW—Freiburg	Ionization chamber	125
Semiflex T31021	PTW—Freiburg	Ionization chamber	70
PinPoint T31014	PTW—Freiburg	Ionization chamber	15
PinPoint 3D 31022	PTW—Freiburg	Ionization chamber	16
Diode P T60017	PTW—Freiburg	Semiconductor detector	0.030
Diode E T60016	PTW—Freiburg	Semiconductor detector	0.030
microDiamond T60019	PTW—Freiburg	Diamond detector	0.004
RAZOR diode	IBA Dosimetry	Semiconductor detector	0.020
EFD 3G‐pSi	IBA Dosimetry	Semiconductor detector	0.160
EDGE detector	Sun Nuclear	Semiconductor detector	0.020
RAZOR chamber	IBA Dosimetry	Ionization chamber	10
CC04	IBA dosimetry	Ionization chamber	40
Exradin W2 1 × 1	Standard Imaging	Plastic scintillator (PSD)	0.785
Exradin W2 1 × 3	Standard Imaging	Plastic scintillator (PSD)	2.356

Finally, a volume‐averaging correction factor ${\left(k_{vol}\right)}_{Q_{0},\;Q_{0}\;}^{f_{msr},\;f_{clin}}$ in clinical and msr field was determined as:

(7) $${\left(k_{vol}\right)}_{Q_{0},\;Q_{0}\;}^{f_{msr},\;f_{clin}}=\frac{{\left(k_{vol}\right)}_{Q_{0}}^{f_{msr,clin}}}{{\left(k_{vol}\right)}_{Q_{0}}^{f_{msr}}}\;$$

A contribution of a volume averaging to a field output correction factor was determined as:

(8) $$k\;=\frac{{\left(k_{vol}\right)}_{Q_{0},\;Q_{0}\;}^{f_{msr},\;f_{clin}}}{k_{Q_{0},\;Q_{0}\;}^{f_{msr},\;f_{clin}}}\;$$

## 3 RESULTS

**FIGURE 3:**
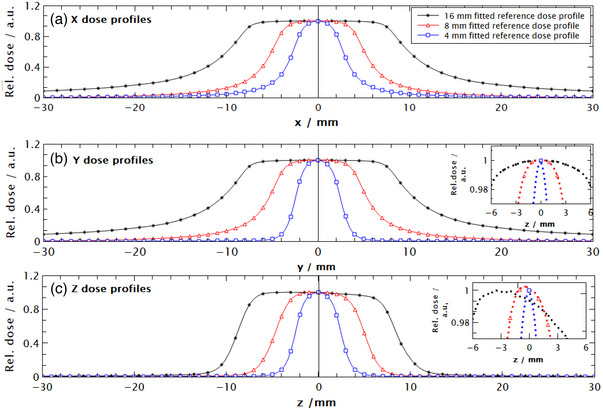
Fitted normalized dose profiles for a 16, 8, and 4 mm field size on GK Perfexion. Data were calculated using Monte Carlo for a shot at the centre of a spherical phantom with a radius of 80 mm. (a) Dose profile on x‐axis, (b) dose profile on the y‐axis with insert showing symmetry in y dose profile, and c) dose profile on the z‐axis with insert of enlarged centre of the profile showing asymmetry. Normalized dose profiles on the x and y major axis are fitted to only positive values, that is, they are considered perfectly symmetrical.

The fitting with proposed fit functions was performed in CurveExpert Professional (Hyamas Development, USA) in a way that all fit functions, nine in total, had a coefficient of determination (R^2^) larger than 0.999. A fitting score, which reflects how closely the model adheres to underlying data, was above 995 (out of 1000) for all fit functions, and the results can be seen in Figure 3.

**FIGURE 4:**
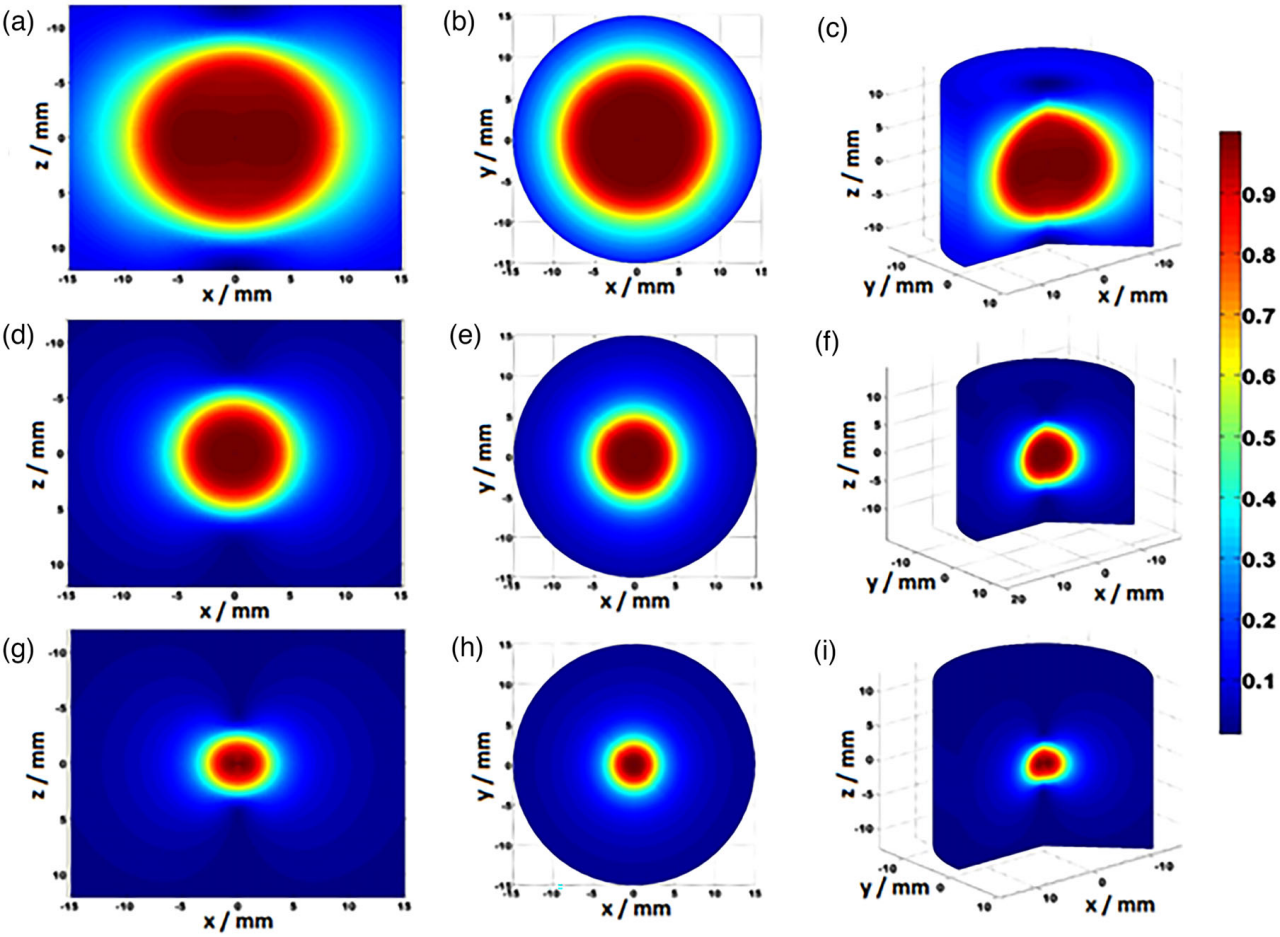
Gamma Knife Perfexion field size simulation using the elliptical model in MATLAB. From (a)–(c). XZ, XY plane, and 3D simulation of 16 mm field size, from (d)–(f). XZ, XY plane, and 3D simulation of 8 mm field size, from (g)–(i). XZ, XY plane, and 3D simulation of 4 mm field size. For the XZ plane y = 0, and z = 0 for the XY plane. In the XZ plane, the field is elliptical due to the z‐dose profile. In the XY plane, the field is circular in shape.

With adequate fit functions and elliptical dose model formalism, coding was performed in MATLAB. Simulations of a single shot for all field sizes can be seen in Figure 4.

**FIGURE 5:**
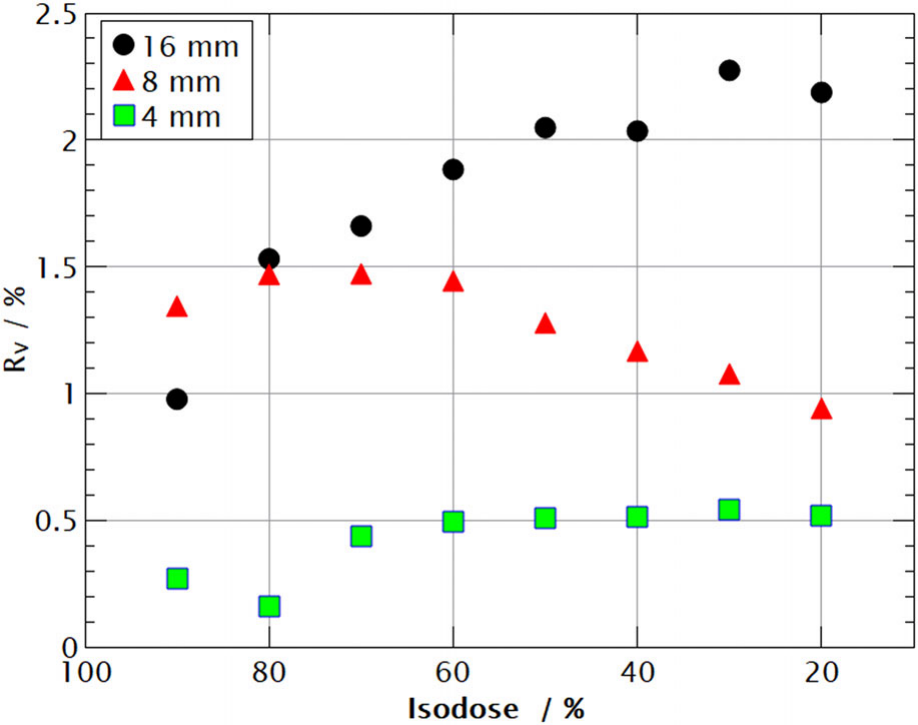
A difference in an absorbed dose calculated by the LGP and the model for different ellipsoid volumes beginning at the isocenter and ending at the givens dose value, for example, 100%−90%, 100%−80%, …, 100%−20%.

The accuracy of a model with respect to an absorbed dose was the highest for the 4 mm field, with the 16 mm field having the largest difference R_v_ from the LGP (Figure 5). For the 4 mm field size, the largest difference was around 0.5%. The difference in the absorbed dose for the 8 mm field is under 1.5% for all examined volumes. For the 16 mm field, the difference is the smallest at the 90% isodose volume and it is gradually increased to 2.25% for the 30% isodose volume.

**FIGURE 6:**
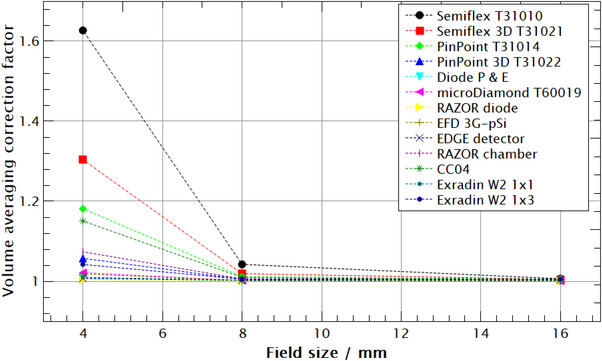
Volume averaging correction factors ${\left(k_{vol}\right)}_{Q_{0}}^{f_{msr,clin}}$ in clin and msr fields for different detectors on Leksell Gamma Knife Perfexion. Diode P T60016 and Diode E T60017 are represented by one data point since their active volumes are identical therefore volume averaging correction factor is the same. Dashed lines are added for better graph readout and should not be interpreted as interpolation of data points.

Volume averaging correction factors for detectors listed in Table 1 are calculated using Equation (1). Since the analytical expression of an absorbed dose is not integrable, Simpson's method of numerical integration was used with one hundred steps in each spatial direction. This resolution, that is, voxel size, provides accurate results up to the third decimal point while limiting volume averaging calculation time. The integration limits are determined using detector schematics provided by the manufacturer. The results of a calculation of volume averaging in clin and mrs fields ${\left(k_{vol}\right)}_{Q_{0}}^{f_{msr,clin}}$ for the studied detectors are shown in Figure 6, and are listed in Table 2.

**TABLE 2:** A result of volume averaging correction factors ${\left(k_{vol}\right)}_{Q_{0}}^{f_{msr,clin}}\;$for different detectors in different fields of Gamma Knife Perfexion, and volume averaging ratios in clinical and machine‐specific reference fields ${\left(k_{vol}\right)}_{Q_{0},\;Q_{0}\;}^{f_{msr},\;f_{clin}}$.

	${\left(k_{vol}\right)}_{Q_{0}}^{f_{msr,clin}}$			${\left(k_{vol}\right)}_{Q_{0},\;Q_{0}\;}^{f_{msr},\;f_{clin}}$		
Detector name	16 mm	8 mm	4 mm	16 mm	8 mm	4 mm
Semiflex T31010	1.006	1.042	1.627	1.000	1.036	1.617
Semiflex 3D T31021	1.005	1.019	1.304	1.000	1.014	1.298
PinPoint T31014	1.005	1.011	1.181	1.000	1.006	1.175
PinPoint 3D T31022	1.003	1.010	1.101	1.000	1.001	1.091
Diode P T60016	1.001	1.000	1.008	1.000	0.999	1.007
Diode E T60017	1.001	1.000	1.008	1.000	0.999	1.007
microDiamond T60019	1.000	1.002	1.021	1.000	1.001	1.020
RAZOR diode	1.001	1.000	1.004	1.000	0.999	1.003
EFD 3G‐pSi	1.001	1.001	1.018	1.000	1.000	1.017
EDGE detector	1.004	1.003	1.007	1.000	0.999	1.004
RAZOR chamber	1.003	1.006	1.074	1.000	1.003	1.071
IBA CC04	1.007	1.014	1.156	1.000	1.007	1.147
Exradin W2 1 × 1	1.003	1.002	1.010	1.000	0.999	1.007
Exradin W2 1 × 3	1.005	1.005	1.042	1.000	1.000	1.037

Detectors whose volume averaging correction factors are marked with red color are not recommended for dosimetry at that field size according to TRS‐483 CoP.

A contribution of volume averaging correction factor ${\left(k_{vol}\right)}_{Q_{0},\;Q_{0}\;}^{f_{msr},\;f_{clin}}$ in total field output correction factor or $k_{Q_{0},\;Q_{0}\;}^{f_{msr},\;f_{clin}}$ was calculated using Equation (8) for detectors that are listed in TRS‐483 CoP.

## 4 DISCUSSION

In this work, an elliptical dose model was used to model different fields created by the narrow photon beams of Gamma Knife Perfexion to determine the volume averaging correction factors for different detectors. An analytical expression for relative dose was obtained with proposed fit functions and an elliptical dose model. All fit functions have shown a high degree of agreement with the normalized dose profiles having R^2^ ≥ 0.999. They are simple to manipulate and, unlike previous functions, don't require additional modification to account for the tilted shape of a z‐axis dose profile. Since dose profiles on the ellipse x and y major axis are almost identical, the same fit function was used, and only the positive axis values were considered. In contrast to the z‐dose profile where it was necessary to use different functions due to their difference in shape and they become less complex as we decrease the field size which can be explained by the increase in the symmetry of the z‐dose as field size decreased (Figure 3c). It is worth noting that for 16 and 8 mm fields, it was necessary to use the sum of five error functions while for 4 mm the sum of two error functions was sufficient. From our analysis, the proposed functions in this paper can be used to describe referent dose profiles.

To calculate volume averaging correction factors, the elliptical dose model must be examined when it comes to an absorbed dose calculation. This was checked for different volumes ranging from the center of dose distribution to given isodose and compared with the data calculated by the LGP (Figure 5). For all investigated volumes, the maximum difference was less than 2.25%, and it decreased with a decrease in field size, for example, for 4 mm field size difference was around 0.5%. The increase in accuracy with decreasing field size is likely due to the shape of the z‐dose profile whose asymmetry is most pronounced for a 16 mm field size. For the 4 mm field, the z‐dose profile is almost symmetrical, therefore it was the easiest to fit, which increased the model's accuracy. Our analysis confirmed that despite the asymmetry of a z‐dose profile, the model is successful in predicting the absorbed dose for all field sizes and can be used for calculating volume‐averaging correction factors.

**FIGURE 7:**
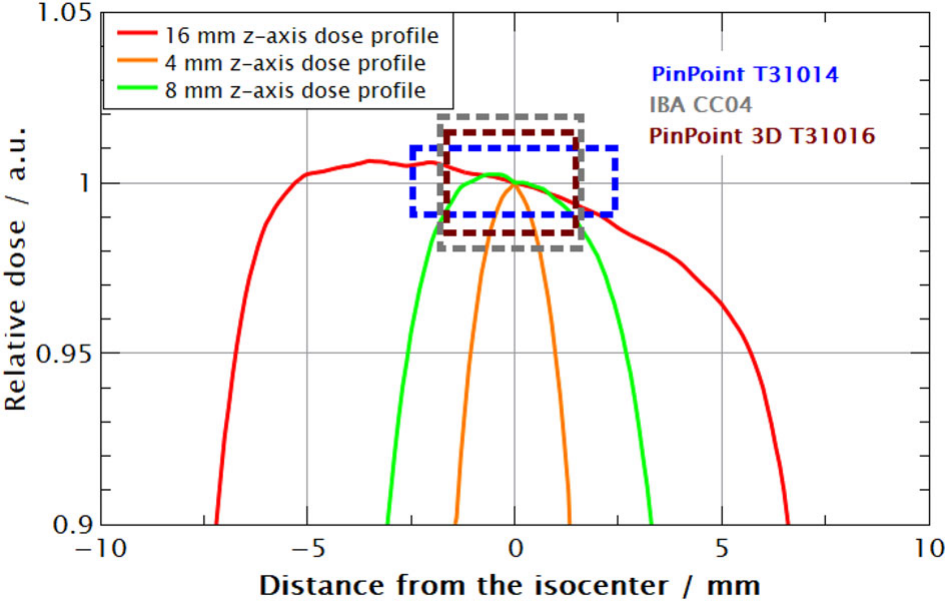
The relative dose profiles on the z‐axis with geometries of three detectors. The gradient on the z‐axis more influences the longer detector increasing the volume averaging correction factor. Dose profiles are normalized at z = 0 mm.

Using numerical integration and Equation (1), the volume averaging correction factor was determined for different detectors due to the lack of such data in TRS‐483 CoP for GK fields.^13^ When calculating volume averaging correction factors, any deviations from the ideally responsive detectors are ignored. It is assumed that all detectors are perfectly isotropic in response and their weighting functions are set to equal unity. Due to the symmetrical distribution of Co‐60 sources around the device's z‐axis, any directional dependence that diode detectors are known off can be omitted in studying volume averaging effect.^21^ In general, volume averaging decreases with the increase of the field size and a decrease in the detector's volume which is expected. However, there are a few examples that need to be addressed. Semiflex T31010 has the largest volume averaging correction factor for all field sizes due to its large volume (V = 125 mm^2^), followed by Semiflex 3D T31021 (V = 70 mm^3^); 1.006, 1.042, 1.627, and 1.005, 1.019, 1.304 for 16, 8, and 4 mm field, respectively. Although it has a larger volume, PinPoint 3D T31022 has lover volume averaging correction than PinPoint T31014 (16 mm^3^ vs. 15 mm^3^) for all field sizes. This is due to the geometry of these chambers where PinPoint T31014 is longer and narrower, and PinPoint 3D T31022 is more compact. A length‐to‐diameter ratio R, is 2.5 and 1.23 for PinPoint and PinPoint 3D, respectively. Positioning the detector with its long axis in z direction makes it more influenced by the asymmetry, and dose gradient of the z‐dose profile over its volume, therefore, increasing the volume averaging correction factor (Figure 7). Importantly, the z‐dose profile also has the lowest FWHM value, that is, it is the shortest (Figure 4). By contrast, at the radial dimensions of these chambers, x, and y—dose profiles are flatter limiting their contribution to the volume averaging correction factor. The larger volume averaging correction factor at the 16 mm field compared with the 8 mm field for some detectors contributed to the lower accuracy of the model for that field size (Figure 5) with a difference of around 0.1% (Table 2). For a 4 mm field size, IBA CC04 has volume averaging comparable with PinPoint although its effective volume is almost three times larger (Table 2). Again, this is due to the longer active volume in the z‐direction of PinPoint T31014 than IBA CC04 (5 mm compared to 3.6 mm) as shown in Figure 7, that is, IBA CC04 is a more compact chamber with an active length to diameter ratio equal to 0.9.

RAZOR chamber is the most compact ionization chamber in this study with an active volume of 10 mm^3^ and, as a result, it has the smallest volume averaging correction factor out of all ionization chambers for all field sizes.

Due to the small size of an effective volume and consequently smaller volume averaging effect semiconductor detectors are the ideal type of detectors for dosimetry in small fields.^5^ This fact is supported by this study for a large majority of explored semiconductor detectors. The single exception is the EFD diode which has a relatively large volume for a semiconductor detector (0.16 mm^3^), therefore the correction factor for the 4 mm field size is 1.018. Diode E T60016 and Diode P T60017 have equal volume averaging correction factors since their active volumes are identical, and the only difference is shielding for the filtration of low‐energy photons. The microDiamond T60019 is characterized by the smallest volume out of all studied detectors; however, its correction factor for the 4 mm field is relatively large being 1.021. This is due to the shape of its effective volume. It is unique in a way that it is a very thin (1 μm) disk with a large diameter of 2.2 mm. This design makes it more sensitive to dose gradients in the XY plane which starts to be noticeable at a 4 mm field increasing the correction for volume averaging. From this, one can see that although the magnitude of the detector's sensitive volume is important, when it comes to dosimetry of GK narrow photon beams, the shape of that volume also has an important role. The manufacturer denotes plastic scintillator detectors Exradin W2 1 × 1 and 1 × 3 as perturbation‐free detectors since they are tissue–equivalent. However, the effect of volume averaging could not be neglected for them (Table 2). In addition, following IAEA TRS‐483 CoP we have selected detectors that are not suitable for dosimetry of Gamma Knife Perfexion photon beams according to their volume averaging correction factor. Therefore, Semiflex T31010, Semiflex 3D T31021, PinPoint T31014, PinPoint 3D T31022, CC04, and RAZOR chamber are not recommended for dosimetry in 4 mm field since their volume averaging correction factor is larger than 1.05 (Table 2). Finally, we have calculated the contribution of the volume averaging correction factor in the field output correction factor for detectors listed in TRS‐483 CoP. For all studied detectors, volume averaging is a dominant perturbation (Table 3). For the semiconductor detectors, the volume averaging contribution is larger than the total field output correction factor. This is expected since they over‐respond to low energy scattered photons and their total correction factors are lower than 1.000 due to the fluence perturbations of these detectors.

**TABLE 3:** Field output correction factors $k_{Q_{0},\;Q_{0}\;}^{f_{msr},\;f_{clin}}$ from IAEA TRS‐483 CoP and contribution of volume averaging correction factor to a total correction.

	$k_{Q_{0},\;Q_{0}\;}^{f_{msr},\;f_{clin}}$			*k*		
Detector name	16 mm	8 mm	4 mm	16 mm	8 mm	4 mm
Semiflex T31010	1.004	N.A.	N.A.	0.996	N.A.	N.A.
PinPoint T31014	1.000	1.030	N.A.	1.000	0.984	N.A.
Diode P T60016	1.000	0.981	0.965	1.000	1.018	1.044
Diode E T60017	1.000	0.996	0.985	1.000	1.003	1.022
microDiamond T60019	1.000	1.005	0.993	1.001	0.996	1.029
IBA CC04	1.021	N.A.	N.A.	0.979	N.A.	N.A.

N.A. stands for not available.

## 5 CONCLUSION

This study used an elliptical dose model of Gamma Knife Perfexion to determine volume averaging correction factors for different detectors used for the dosimetry of Gamma Knife narrow photon beams. With newly introduced fit functions for relative dose profiles, an analytical expression for 3D relative dose distribution was obtained and a simulation of an absorbed dose model was created in MATLAB. The accuracy of a model was confirmed against LGP with the respect to the calculated absorbed dose for a given ellipsoid volume with very good agreement for all field sizes.

Volume averaging correction factors were determined for fourteen detectors in three available field sizes. This was done by numerical integration of normalized absorbed dose distribution over the detector's volume. Generally, the volume averaging correction factor increases as filed size decreases. Observed deviations are likely due to the limitations of an elliptical dose model, primarily in fitting the z‐axis dose profile. Finally, using IAEA TRS‐483 CoP formalism we have shown that volume averaging has a dominant contribution to the field output correction factor and have recommended detectors suitable for dosimetry of Gamma Knife Perfexion photon beams based on the volume averaging criteria.

## AUTHOR CONTRIBUTIONS

All authors have contributed equally and significantly to the manuscript.

## CONFLICT OF INTEREST STATEMENT

No conflict of interest.

## Supporting information


Supporting Information

Supporting Information

Supporting Information

Supporting Information

Supporting Information

Supporting Information

Supporting Information
