# Supplementary figures and images for: Determination of volume averaging correction factors using an elliptical absorbed dose model for Gamma Knife Perfexion
Source: J Appl Clin Med Phys. 2023 Aug 25;24(10):e14109. doi: 10.1002/acm2.14109 (PMC10562043; doi:10.1002/acm2.14109)

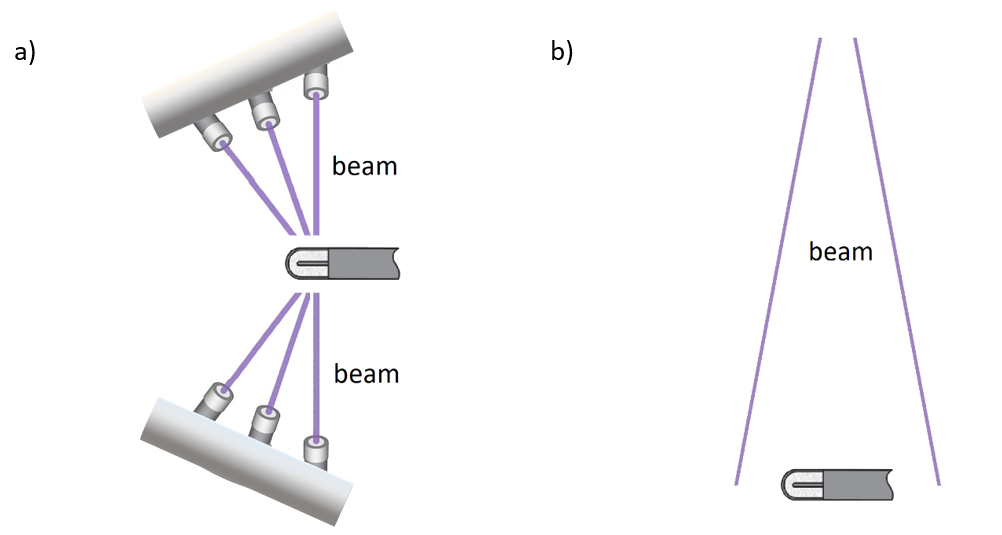

Supplement: Supplementary file 1 — Supporting Information [file ACM2-24-e14109-s007.png]

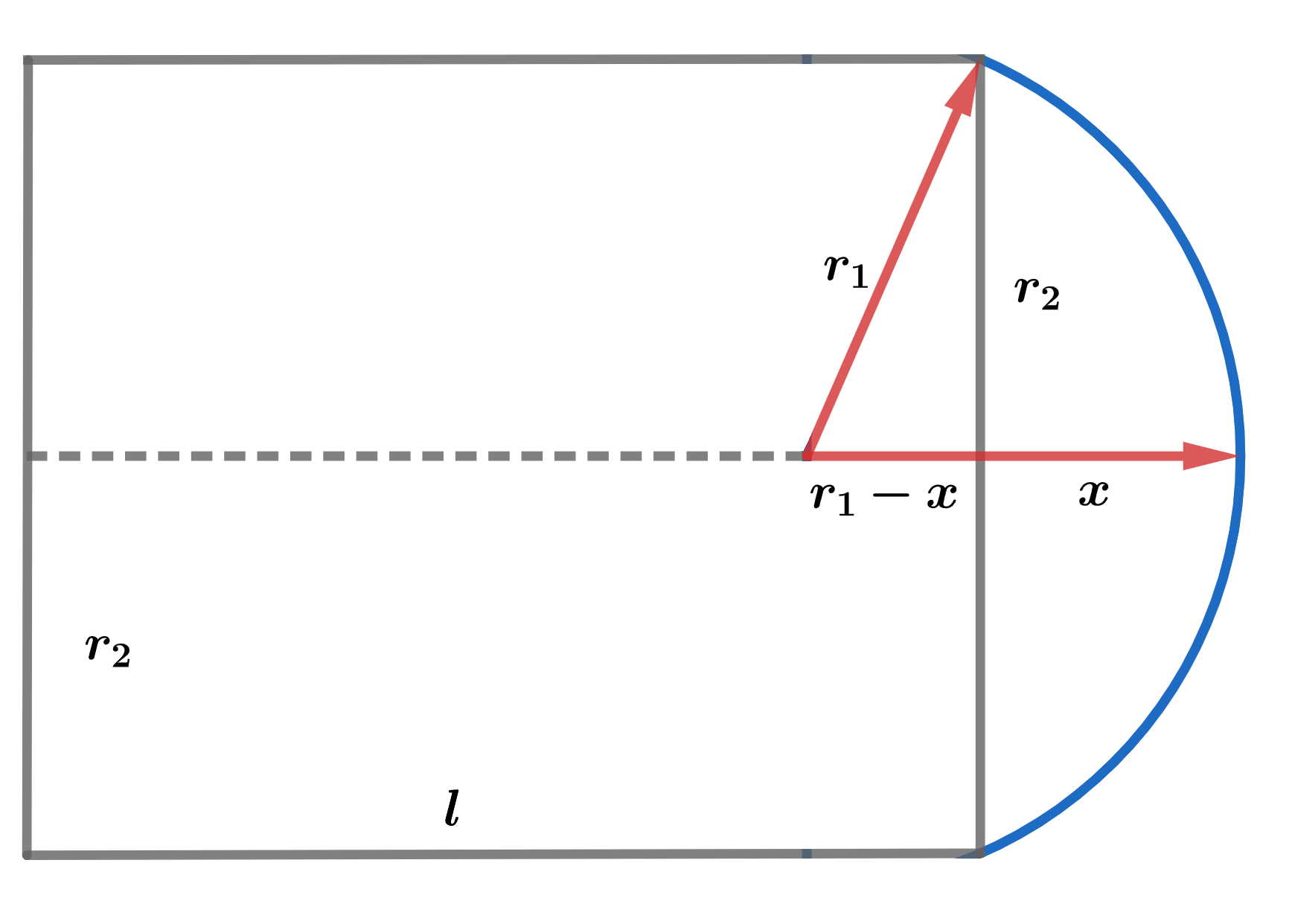

Supplement: Supplementary file 2 — Supporting Information [file ACM2-24-e14109-s002.png]

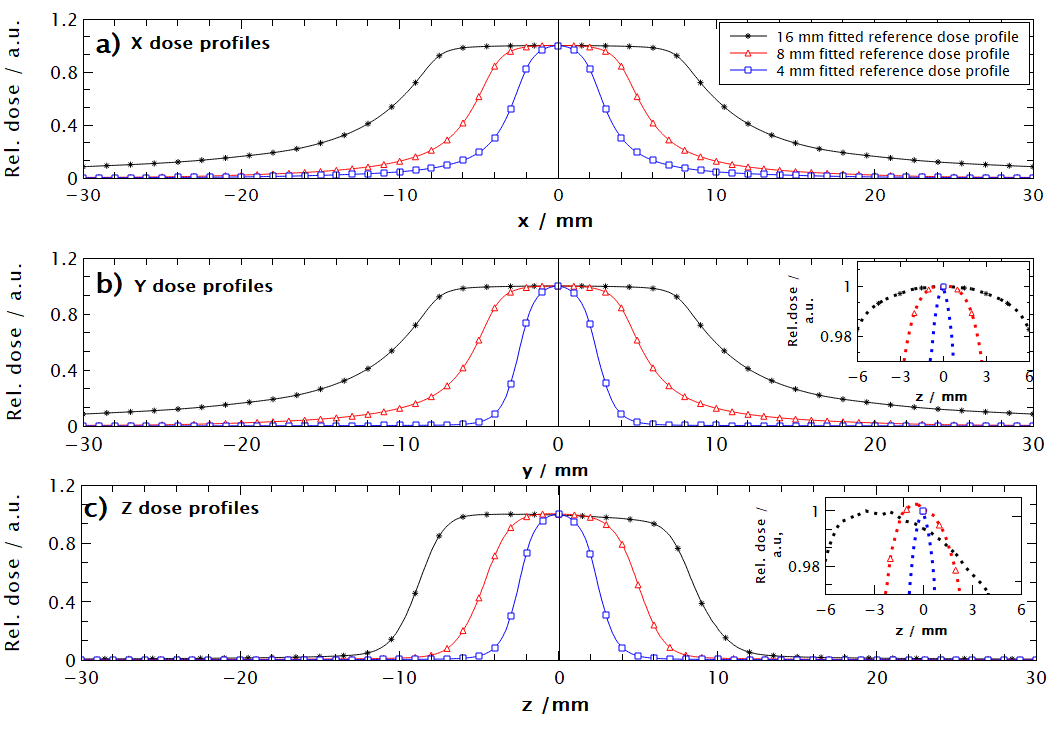

Supplement: Supplementary file 3 — Supporting Information [file ACM2-24-e14109-s004.png]

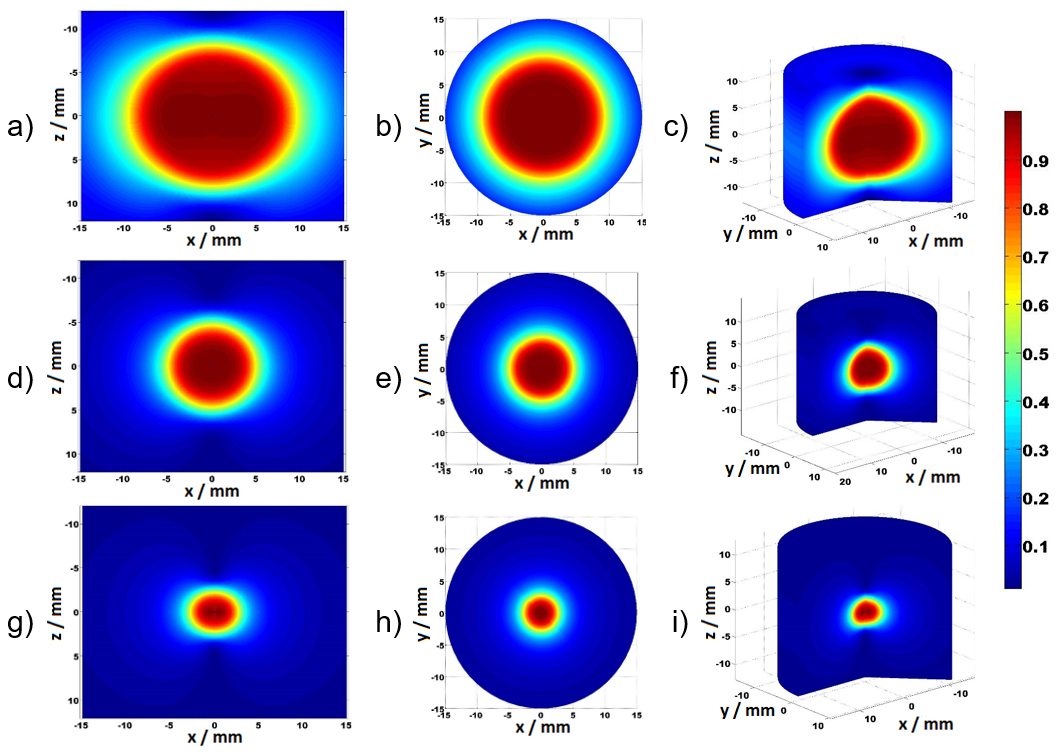

Supplement: Supplementary file 4 — Supporting Information [file ACM2-24-e14109-s006.png]

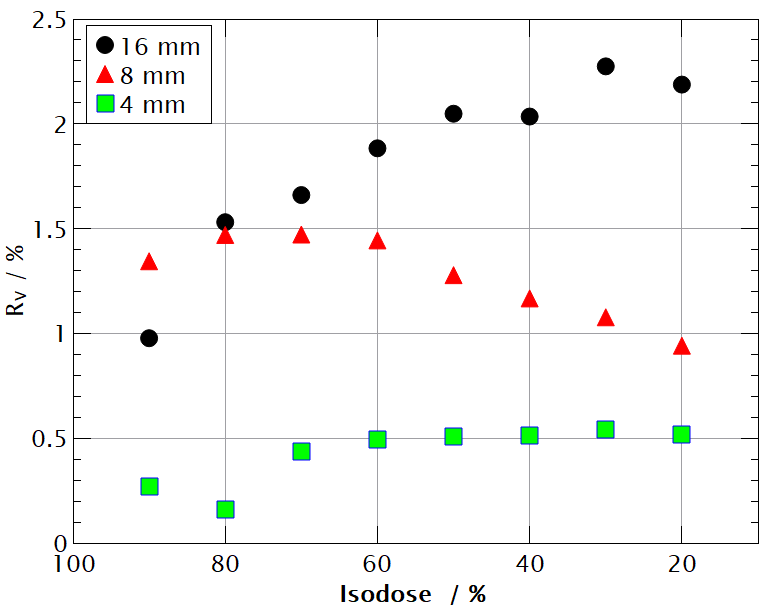

Supplement: Supplementary file 5 — Supporting Information [file ACM2-24-e14109-s001.png]

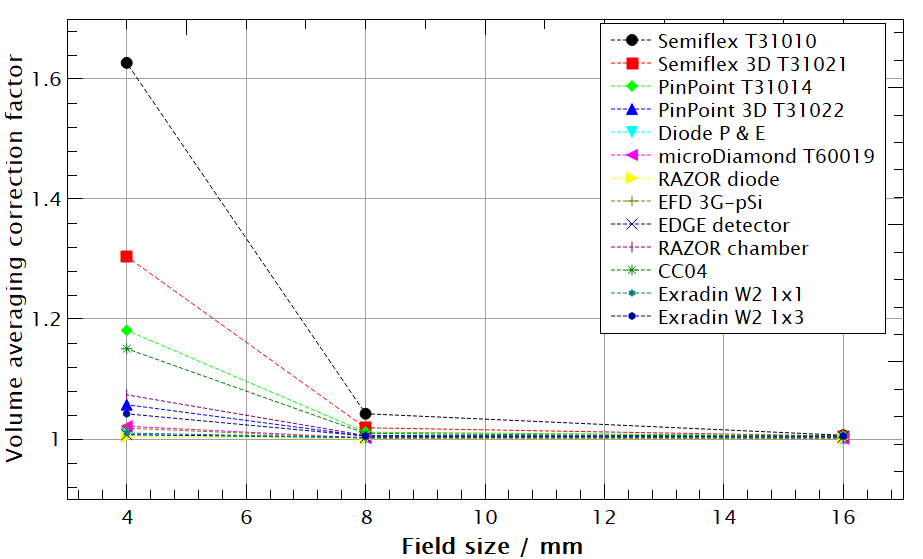

Supplement: Supplementary file 6 — Supporting Information [file ACM2-24-e14109-s005.png]

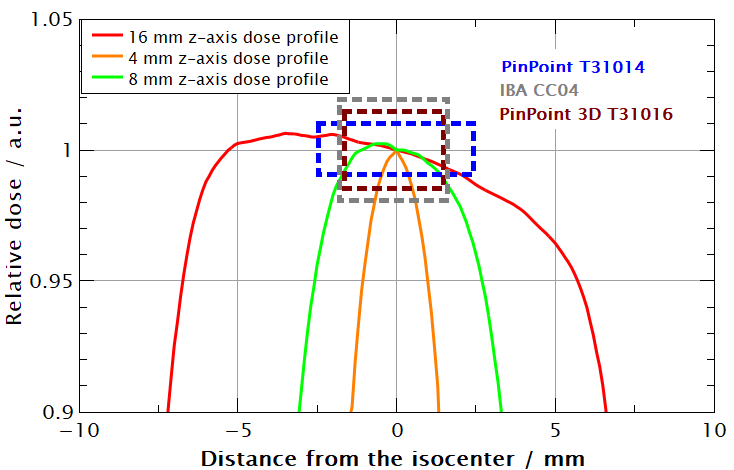

Supplement: Supplementary file 7 — Supporting Information [file ACM2-24-e14109-s003.png]
